# Supplementary material for: The Effects of Rumen-Protected Choline and Rumen-Protected Nicotinamide on Liver Transcriptomics in Periparturient Dairy Cows
Source: Metabolites. 2023 Apr 26;13(5):594. doi: 10.3390/metabo13050594 (PMC10222217; doi:10.3390/metabo13050594)
Supplement: Supplementary file 1 [file metabolites-13-00594-s001.zip › metabolites-2340017-supplementary.pdf]

Supplementary Materials

Liver Sample Acquisition and Storage

Shave off the area at the junction of the connecting line from the 10th and 11th ribs and hip joints to the elbow joint of the right forelimb, sterilize with alcohol, and anaesthetize with 2mL lidocaine hydrochloride subcutaneously. After 5min, evaluate the anaesthetic effect through skin reaction. Use a knife to cut the body wall, insert a biopsy needle into the liver, collect about 100mg of tissue, quickly freeze it in liquid nitrogen, and store it at - 80 ° C for testing.

The Quality Control of Sequencing Data

Convert the sequenced image signal into text signal through CASAVA base calling, and store it in fastq format as the original data. Distinguish the data of each sample according to the index sequence, filter the original sequenced data, so as to obtain high-quality sequenced data (clean data) to ensure the smooth progress of subsequent analysis. The specific steps and sequence are as follows: (1) Remove the connector sequence in reads, Remove the reads that are not inserted due to self connection of the connector and other reasons; (2) Trim the base with low quality (mass value less than 30) at the end of the sequence (3 'end). If there are still bases with mass value less than 10 in the remaining sequence, remove the whole sequence, otherwise keep it; (3) Remove the reads with the proportion of N more than 10%; (4) Discard the sequences whose length is less than 50bp after removing adapter and quality trimming.

Use software: fastp. After the quality cutting is completed, the statistics and quality evaluation of the quality control data shall be conducted again, including: (1) A/T/G/C base content distribution statistics; (2) Base quality distribution statistics; (3) Base error rate distribution statistics.

It is recommended to add connector information:

5’：AGATCGGAAGAGCACACGTC；

3‘：AGATCGGAAGAGCGTCGTGT；

The clean data were obtained by removing adapters, low-quality reads, and reads containing poly-N from the raw data. The Q20 and Q30 values, GC content, and sequence duplication levels were calculated for the clean data. The clean data were used for further analysis.

The Analysis of Gene Expression

Use RSEM to obtain the Read Counts of each sample gene/transcript using the results of genome comparison and genome annotation files. Then TPM (Transcripts Per-Million reads) transformation was performed to obtain standardized gene/transcript expression level.

Supplementary Table

Table S1 Statistics for quality control of sequencing data.

Raw reads: the total number of the original sequencing data; Clean reads: total number of sequenced data after quality control; Error rate (%): the average error rate of sequencing base corresponding to quality control data is generally below 0.1%; Q20 (%) and Q30 (%): evaluate the quality of sequencing data after quality control. Q20 and Q30 refer to the percentage of bases with sequencing quality above 99% and 99.9% in total bases respectively (generally, Q20 is above 85% and Q30 is above 80%); GC content (%): the percentage of the total G and C bases corresponding to the quality control data in the total base group.

Sample name*	Raw reads	Clean reads	Error rate (%)	Q20 (%)	Q30 (%)	GC content (%)
RPC1	46393804	43667416	0.0258	97.64	93.57	49.97
RPC2	49012014	45527436	0.0241	98.43	95.18	46.70
RPC3	46387460	43829246	0.0254	97.78	93.92	50.21
RPC4	49396522	44113370	0.0258	97.66	93.58	48.06
RPC5	46936474	42322130	0.0253	97.80	94.02	49.35
RPM1	61459658	61459658	0.0244	98.27	94.81	46.51
RPM2	48306010	48306010	0.0253	97.85	94.02	49.93
RPM3	53453998	53453998	0.0263	97.51	93.02	45.73
RPM4	49033338	49033338	0.0259	97.65	93.47	49.79
RPM5	48566316	48566316	0.0263	97.49	93.08	49.83

*RPC = rumen protected choline group; RPM = rumen protected nicotinamide group.

Table S2 Differential gene statistics of up and down regulation.

In order to control the probability or frequency of errors in the overall inference results, BH (FDR correction with Benjamin/Hochberg) method is used to conduct multiple tests to correct the *P*-value obtained from statistical tests. The corrected *P*-value is *P*-adjust. The DES eq2 software based on negative binomial distribution was used to analyze Raw Counts, and genes/transcripts with different expression between groups were obtained based on certain standardized processing and screening conditions. Differential gene screening parameters: *P* -adjust<0.05 & |log2FoldChange|⩾1.

Treatments*	all	up	down
RPC vs RPM	1123	640	483

*RPC = rumen protected choline group; RPM = rumen protected nicotinamide group.

Supplementary Figure

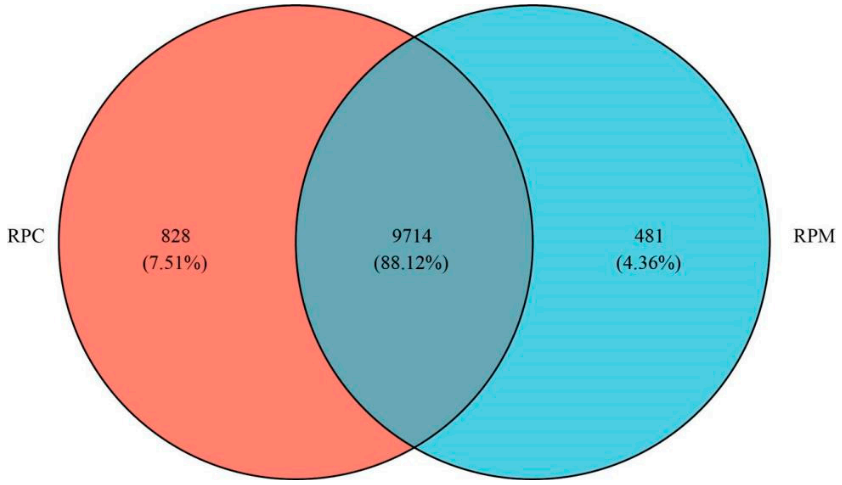

Figure S1 Venn analysis between RPC and RPM group.

RPC = rumen protected choline; RPM = rumen protected nicotinamide.
